# Integrated Multidisciplinary Approach to Acute Chest Pain: Perspectives From Family Medicine, Emergency Medicine, and Internal Medicine

**DOI:** 10.7759/cureus.74423

**Published:** 2024-11-25

**Authors:** Amna Yaseen, Huzaifa Kamran Khan, Areeb Asghar, Zahoor Ahmad, Talha Masood, Muhammad Hamza Ghufran, Tajala Fayyaz, Muzzamil Samad, Ahmad Amin, Salman Wali, Naqeeb Ullah, Sundas Safdar, Mahwash Nazir, Hanifullah Hanfi, Tamanna Nazir

**Affiliations:** 1 General Practice, Hayatabad Medical Complex, Peshawar, PAK; 2 Medicine, Foundation University School of Health Sciences, Islamabad, PAK; 3 Emergency Medicine, Government Mian Meer Hospital, Lahore, PAK; 4 Internal Medicine, Kuwait Teaching Hospital, Peshawar, PAK; 5 Internal Medicine, Northern Lincolnshire and Goole NHS Foundation Trust, Scunthorpe, GBR; 6 Internal Medicine, Lady Reading Hospital, Peshawar, PAK; 7 Cardiology, Mardan Medical Complex, Mardan, PAK; 8 Internal Medicine, Mardan Medical Complex, Mardan, PAK; 9 Radiology, Lady Reading Hospital, Peshawar, PAK; 10 Internal Medicine, Borders General Hospital, Melrose, GBR; 11 Internal Medicine, Hayatabad Medical Complex, Peshawar, PAK

**Keywords:** acute chest pain, emergency medicine, family medicine, internal medicine, multidisciplinary approach

## Abstract

Background: Acute chest discomfort is a common clinical problem that has to be well understood and managed collaboratively by specialists from many fields of medicine.

Objective: This study aimed to explore and evaluate the perspectives of healthcare professionals in family, emergency, and internal medicine regarding the management of acute chest pain, with a specific focus on diagnostic practices, interdisciplinary collaboration, and protocol adherence to establish best practices for a unified approach.

Methodology: This cross-sectional study, conducted from June 2022 to July 2024, included 218 healthcare professionals with over a year of experience in family, emergency, and internal medicine, selected through convenient sampling from hospitals such as Lady Reading Hospital, Hayatabad Medical Complex, Mardan Medical Complex, and Government Mian Meer Hospital. Data was collected through structured questionnaires covering demographics, clinical protocols, inter-disciplinary communication, and management challenges, complemented by semi-structured interviews for deeper insights. Statistical analysis was performed using SPSS version 26, with chi-square tests comparing responses across specialties, considering p < 0.05 as statistically significant.

Results: The results showed that emergency medicine practitioners had the highest use of diagnostic tools, with 80 out of 80 (100%) using electrocardiogram (ECG) and 78 out of 80 (97.5%) using troponin tests, compared to 60 out of 70 (85.71%) and 40 out of 70 (57.14%) in family medicine (p < 0.001 for both). Additionally, 70 out of 80 (87.5%) in emergency medicine reported time constraints affecting management. Communication barriers were noted by 50 out of 80 (62.5%) in emergency medicine and 45 out of 70 (64.29%) in family medicine. Interdisciplinary collaboration was reported at 50 out of 70 (71.43%) in family medicine and 60 out of 80 (75%) in emergency medicine. A lack of standardized protocols was especially high in internal medicine, impacting 65 out of 68 (95.59%) participants (p<0.001).

Conclusion: Emergency medicine professionals consistently utilize diagnostic tools like ECGs and troponin tests more frequently, reflecting their time-sensitive clinical environment, but also report significant time constraints. In contrast, internal medicine practitioners, who generally have more years of experience, reported the highest adherence to management protocols, yet they also identified a lack of standardized guidelines as a major barrier. Family medicine professionals, while showing strong interdisciplinary collaboration, had lower utilization rates of advanced diagnostic tools, which may impact early decision-making. These disparities underline the need for unified protocols and improved communication pathways across specialties to enhance diagnostic accuracy and streamline management.

## Introduction

One frequent clinical manifestation that may be difficult to diagnose and treat in various healthcare settings is acute chest discomfort [1,2]. It often signals the presence of dangerous underlying illnesses that need immediate assessment and treatment, such as acute coronary syndrome, pulmonary embolism, or aortic dissection [3]. For the best possible patient results, an integrated multidisciplinary strategy including insights from internal, emergency, and family medicine is necessary, given the variety of etiologies linked to chest pain [4].

For many patients with acute chest pain, family medicine practitioners are often the first to assess them. They typically conduct the initial evaluation and decide if the patient needs to be quickly referred to a specialist for further treatment. In this process, they play a vital role in assessing risk and identifying non-cardiac causes, such as gastrointestinal or musculoskeletal issues, which may present with symptoms similar to heart disease [5,6]. Emergency medicine professionals are responsible for expedient triage, stabilization, and sophisticated diagnostic assessments in the interim [7]. Various diagnostic techniques, like imaging investigations, laboratory tests, and electrocardiograms, quickly differentiate between life-threatening illnesses and less urgent presentations [8]. They do this by using critical decision-making abilities.

When a patient presents with acute chest discomfort, internal medicine specialists are essential to their therapy and follow-up care [9]. They are in charge of treating chronic risk factors that might lead to repeated bouts of chest discomfort, interpreting diagnostic data, and arranging treatment among experts [10]. Working together, these three disciplines improve diagnostic accuracy and provide a thorough awareness of the patient's health history, socioeconomic determinants of health, and possible treatment obstacles [11].

Effective therapy for patients with acute chest pain may be hampered by a lack of integrated care pathways and consistent communication despite the expertise and experience each specialty brings to the table. Patients presenting to family Medicine clinics may be self-triaging to a lower acuity setting and often have a lower pretest probability for acute conditions like acute coronary syndrome, pulmonary embolism, or aortic dissection compared to patients in emergency medicine or inpatient settings. This difference in patient acuity may impact diagnostic pathways and management decisions. To identify best practices for coordinated care, this study examines and evaluates the viewpoints of healthcare professionals from internal medicine, emergency medicine, and family medicine on managing acute chest pain, focusing on demographic characteristics, diagnostic tool usage, adherence to clinical protocols, communication dynamics, and perceived challenges. The goal is to uncover practice gaps and opportunities for enhancing interdisciplinary collaboration and protocol standardization to optimize patient outcomes.

## Materials and methods

Study design and setting

This cross-sectional study, conducted from June 2022 to July 2024 across multiple hospitals in Pakistan, surveyed 218 family, emergency, and internal medicine healthcare professionals on managing acute chest pain, using structured questionnaires and semi-structured interviews.

Inclusion and exclusion criteria

Healthcare professionals (physicians and specialists) employed in family, emergency, and internal medicine were eligible for inclusion in the study. "Physicians" were defined as individuals holding a medical degree (MBBS) who are still in training or early in their careers. "Specialists" were defined as licensed medical doctors who have completed postgraduate training and certification in their respective fields and hold titles such as "Consultant" or "Specialist". Specialists generally have more years of experience compared to physicians and are considered experts in their fields.

Participants were required to have at least one year of clinical experience in their respective specialties and to provide informed consent. Those who did not directly manage patients with acute chest pain declined to participate or failed to provide informed consent were excluded from the study.

In addition to attending physicians and specialists, residents were also included as participants in the study, provided they met the minimum requirement of at least one year of clinical experience. This inclusion aimed to capture a broad range of experiences from both early-career and more experienced professionals.

Sampling method

A convenience sampling method was employed, allowing for the efficient recruitment of healthcare professionals from the selected hospitals. The rationale for using convenience sampling was to capture a diverse and representative sample of healthcare professionals working in different specialties and clinical settings, ensuring a comprehensive perspective on managing acute chest pain. While convenience sampling may introduce selection bias, it was deemed appropriate given the study's objectives and practical constraints, such as time and resource availability. This sampling strategy enabled the inclusion of various participants across specialties, which is critical for examining interdisciplinary collaboration and differences in clinical practices.

Sample size

The study included a total of 218 healthcare professionals selected using a convenience sampling method. This approach was chosen to efficiently capture a broad range of perspectives from physicians and specialists in Family Medicine, Emergency Medicine, and Internal Medicine across multiple hospitals in Pakistan, including Hayatabad Medical Complex, Peshawar, Lady Reading Hospital, Peshawar, Mardan Medical Complex, Mardan, and Government Mian Meer Hospital, Lahore. Participants included attending physicians, specialists (consultants), and residents, all with at least one year of clinical experience in their respective fields and who provided informed consent. Specialists were defined as physicians who had completed postgraduate training and were fully certified in their areas of expertise. The study excluded those who did not directly manage patients with acute chest pain, those who declined participation, or those who did not provide informed consent. While convenience sampling may introduce some selection bias, it was effective for reaching a diverse sample and ensuring the study captured the multidisciplinary approach to managing acute chest pain. The sample size was deemed sufficient for a comprehensive assessment of the professionals' viewpoints and experiences.

Data collection

Structured questionnaires (Appendices) intended to elicit the opinions of medical professionals on the treatment of acute chest discomfort were used to gather data. Sections on demographic data, clinical procedures, disciplinary communication, and perceived management difficulties were all included in the questionnaire. Furthermore, a subgroup of participants had semi-structured interviews to get further understanding of their perspectives and experiences.

Statistical analysis

SPSS Software version 26 was used for statistical analysis. The questionnaire answers and demographic data were compiled using descriptive statistics. The three fields' differing points of view were compared and contrasted using Chi-square tests. A statistically significant criterion of p<0.05 was used.

Ethical approval

The Institutional Review Board (IRB) granted the research ethical approval. Before being included in the research, all volunteers gave their informed permission, guaranteeing anonymity and the freedom to leave at any moment without facing any consequences.

## Results

The demographic profile of healthcare professionals managing acute chest pain (Table 1), highlights key characteristics across the three specialties: internal medicine (n=68), emergency medicine (n=80), and family medicine (n=70). In terms of age, a significant proportion of family medicine practitioners (30 out of 70, 42.86%) and emergency medicine specialists (30 out of 80, 37.50%) are in their 30s to 40s, while a notable percentage in internal medicine (28 out of 68, 41.18%) are over 50 years. Gender distribution indicates a higher proportion of females in internal medicine (43 out of 68, 63.24%) compared to family medicine (30 out of 70, 57.14%) and emergency medicine (35 out of 80, 56.25%). Regarding years of experience, 30 family medicine practitioners (42.86%) and 40 emergency medicine practitioners (50.00%) report over 10 years of experience, while internal medicine has the largest proportion of professionals with over 10 years of experience (38 out of 68, 55.88%).

**Table 1:** Demographic profile of healthcare professionals involved in acute chest pain management

Characteristic		Family Medicine (n=70)	Emergency Medicine (n=80)	Internal Medicine (n=68)
Age (Years)	<30	10 (14.29%)	5 (6.25%)	5 (7.35%)
	30-40	30 (42.86%)	30 (37.50%)	20 (29.41%)
	41-50	20 (28.57%)	25 (31.25%)	15 (22.06%)
	>50	10 (14.29%)	20 (25.00%)	28 (41.18%)
Gender	Male	40 (57.14%)	45 (56.25%)	25 (36.76%)
	Female	30 (42.86%)	35 (43.75%)	43 (63.24%)
Years of Experience	<5	10 (14.29%)	15 (18.75%)	15 (22.06%)
	5-10	30 (42.86%)	25 (31.25%)	15 (22.06%)
	>10	30 (42.86%)	40 (50.00%)	38 (55.88%)

Clinical practices and communication patterns among professionals across specialties in acute chest pain management (Table 2). ECG usage is universally high, with emergency medicine leading at 100% (80 out of 80), followed by internal medicine at 95.59% (65 out of 68), and family medicine at 85.71% (60 out of 70). Troponin testing is predominantly used by emergency medicine (78 out of 80, 97.50%) in contrast to family medicine (40 out of 70, 57.14%) and internal medicine (60 out of 68, 88.24%). Imaging studies are more frequently utilized in internal medicine (55 out of 68, 80.88%) and emergency medicine (60 out of 80, 75.00%), compared to family medicine (20 out of 70, 28.57%). In management protocols, adherence is high among emergency medicine (70 out of 80, 87.50%) and internal medicine (60 out of 68, 88.24%) professionals but somewhat lower in family medicine (50 out of 70, 71.43%). Regular communication was observed in 50 out of 70 family medicine (71.43%), 65 out of 80 emergency medicine (81.25%), and 55 out of 68 internal medicine practitioners (80.88%). Emergency medicine practitioners cite time constraints as a significant barrier (70 out of 80, 87.50%), with similar responses in family medicine (55 out of 70, 78.57%) and internal medicine (50 out of 68, 73.53%). Notably, a lack of standard protocols was reported by a large proportion of internal medicine professionals (65 out of 68, 95.59%), suggesting a need for improved interdisciplinary alignment.

**Table 2:** Clinical practices and communication dynamics among disciplines in acute chest pain management

Variables		Family Medicine (n=70)	Emergency Medicine (n=80)	Internal Medicine (n=68)
Diagnostic Tools	ECG	60 (85.71%)	80 (100.00%)	65 (95.59%)
	Troponin Tests	40 (57.14%)	78 (97.50%)	60 (88.24%)
	Imaging Studies	20 (28.57%)	60 (75.00%)	55 (80.88%)
Protocols	Management Protocols	50 (71.43%)	70 (87.50%)	60 (88.24%)
	Data Management Protocols	40 (57.14%)	60 (75.00%)	50 (73.53%)
	Triage and Immediate Stabilization	30 (42.86%)	80 (100.00%)	40 (58.82%)
Frequency of Communication	Regularly	50 (71.43%)	65 (81.25%)	55 (80.88%)
	Occasionally	15 (21.43%)	10 (12.50%)	10 (14.71%)
	Rarely	5 (7.14%)	5 (6.25%)	3 (4.41%)
Communication Method	Verbal Updates	55 (78.57%)	65 (81.25%)	60 (88.24%)
	Written Reports	18 (25.71%)	30 (37.50%)	25 (36.76%)
Perceived Challenges	Time Constraints	55 (78.57%)	70 (87.50%)	50 (73.53%)
	Communication Barriers	45 (64.29%)	50 (62.50%)	48 (70.59%)
	Lack of Standard Protocols	50 (71.43%)	60 (75.00%)	65 (95.59%)
	Patient Factors	40 (57.14%)	30 (37.50%)	35 (51.47%)

The perspectives of healthcare professionals on clinical practices and challenges in managing acute chest pain (Table 3) reveal varying levels of confidence and collaboration among the specialties. In clinical decision-making, the majority of emergency medicine practitioners (70 out of 80, 87.50%) expressed confidence, followed by internal medicine (55 out of 68, 80.88%) and family medicine (45 out of 70, 64.29%). Interdisciplinary collaboration was reported highest in internal medicine (60 out of 68, 88.24%), with strong participation from emergency medicine (60 out of 80, 75.00%) and family medicine (50 out of 70, 71.43%). Challenges in management were also prevalent, with emergency medicine reporting the highest (60 out of 80, 75.00%), followed closely by internal medicine (50 out of 68, 73.53%) and family medicine (40 out of 70, 57.14%). Regular communication was predominantly observed in internal medicine (60 out of 68, 88.24%) and emergency medicine (70 out of 80, 87.50%), with family medicine slightly lower (50 out of 70, 71.43%). Training and education needs were noted by emergency medicine practitioners (60 out of 80, 75.00%), and internal medicine (50 out of 68, 73.53%), while family medicine saw a somewhat lower figure (40 out of 70, 57.14%). Collaborative decision-making received strong endorsement from emergency medicine (70 out of 80, 87.50%) and internal medicine (60 out of 68, 88.24%), with family medicine practitioners also highly supportive (50 out of 70, 71.43%).

**Table 3:** Perspectives on clinical practices and challenges in acute chest pain management

Variables	Family Medicine (n=70)	Emergency Medicine (n=80)	Internal Medicine (n=68)
Clinical Decision-Making	45 (64.29%)	70 (87.50%)	55 (80.88%)
Interdisciplinary Collaboration	50 (71.43%)	60 (75.00%)	60 (88.24%)
Challenges in Management	40 (57.14%)	60 (75.00%)	50 (73.53%)
Preferred Communication Methods	50 (71.43%)	70 (87.50%)	60 (88.24%)
Training and Education Needs	40 (57.14%)	60 (75.00%)	50 (73.53%)
Collaborative Decision-Making	50 (71.43%)	70 (87.50%)	60 (88.24%)

A comparative analysis of clinical practices and perceived challenges in acute chest pain management across disciplines (Table 4), underscores the varying approaches among specialties. Use of ECG was universal in emergency medicine (80 out of 80, 100.00%), high in internal medicine (65 out of 68, 95.59%), and substantial in family medicine (60 out of 70, 85.71%), with statistical significance (p=0.002). Troponin testing was markedly more frequent in emergency medicine (78 out of 80, 97.50%) than in internal medicine (60 out of 68, 88.24%) or family medicine (40 out of 70, 57.14%), also with statistical significance (p=0.001). Imaging studies showed a similar pattern, with higher use in internal medicine (55 out of 68, 80.88%) and emergency medicine (60 out of 80, 75.00%) compared to family medicine (20 out of 70, 28.57%) (p=0.003). Adherence to management protocols was highest in internal medicine (60 out of 68, 88.24%) and emergency medicine (70 out of 80, 87.50%), with family medicine at 71.43% (50 out of 70) (p=0.025). Time constraints were perceived as a significant issue by emergency medicine (70 out of 80, 87.50%), family medicine (55 out of 70, 78.57%), and internal medicine (50 out of 68, 73.53%) (p=0.045). Notably, a high proportion of internal medicine professionals (65 out of 68, 95.59%) identified the lack of standard protocols as a challenge, highlighting the need for enhanced interdisciplinary cooperation (p=0.001).

**Table 4:** Comparative analysis of clinical practices and challenges in acute chest pain management across disciplines

Variables	Family Medicine (n=70)	Emergency Medicine (n=80)	Internal Medicine (n=68)	p-value	Chi-square (χ²)
Use of ECG	60 (85.71%)	80 (100.00%)	65 (95.59%)	0.002	10.56
Use of Troponin Tests	40 (57.14%)	78 (97.50%)	60 (88.24%)	0.001	17.34
Use of Imaging Studies	20 (28.57%)	60 (75.00%)	55 (80.88%)	0.003	12.89
Management Protocols	50 (71.43%)	70 (87.50%)	60 (88.24%)	0.025	7.98
Data Management Protocols	40 (57.14%)	60 (75.00%)	50 (73.53%)	0.041	5.66
Regular Communication	50 (71.43%)	65 (81.25%)	55 (80.88%)	0.159	2.21
Preferred Communication Method	50 (71.43%)	70 (87.50%)	60 (88.24%)	0.027	6.59
Perceived Time Constraints	55 (78.57%)	70 (87.50%)	50 (73.53%)	0.045	5.02
Communication Barriers	45 (64.29%)	50 (62.50%)	48 (70.59%)	0.342	1.18
Lack of Standard Protocols	50 (71.43%)	60 (75.00%)	65 (95.59%)	0.001	11.37
Triage and Immediate Stabilization	30 (42.86%)	80 (100.00%)	40 (58.82%)	0.001	14.12
Collaborative Decision-Making	50 (71.43%)	70 (87.50%)	60 (88.24%)	0.03	7.18

## Discussion

The study's conclusions draw attention to significant differences in clinical procedures and challenges faced in managing acute chest pain across specialties, including family medicine, emergency medicine, and internal medicine. Notably, diagnostic tool utilization, such as troponin testing and ECGs, varied among specialties. In this study, 100% of emergency medicine practitioners utilized ECGs, compared to 95.59% in internal medicine and 85.71% in family medicine (p=0.002). This aligns with other studies emphasizing the urgency of rapid cardiac evaluation in emergency departments for potentially life-threatening conditions like myocardial infarction [12]. However, the relatively lower rate of ECG use in family medicine may reflect differences in patient populations and clinical settings, as many patients presenting to family medicine may have non-cardiac causes of chest pain. This suggests that the observed differences might not necessarily indicate a deficiency in early risk assessment but rather a variation in the likelihood of cardiac etiology based on patient presentation.

Furthermore, there were notable differences in the usage of troponin assays; 97.50% of doctors in emergency medicine used this vital biomarker, but only 57.14% in family medicine (p=0.001). This result aligns with previous research emphasizing the critical role of troponin testing in emergency settings to rule out myocardial infarction [13,14]. However, the lower utilization of troponin testing in family medicine may reflect the outpatient nature of this setting, where patients with concerning chest pain are often referred to the emergency department for expedited evaluation and management. This process may help avoid potential delays associated with outpatient testing, ensuring timely care for patients at risk of cardiac events.

Compared to 71.43% of family medicine practitioners, 87.50% of emergency medicine practitioners reported adhering to management procedures (p=0.025). This distinction draws attention to the organized setting of emergency treatment when stricter adherence to acute care procedures is expected. On the other hand, irregularities in patient outcomes and the provision of treatment may result from a failure to follow family medicine management procedures. According to research by Blankstein et al. [15], defined protocols are essential for addressing acute chest discomfort because they help with prompt decision-making and the right kind of referral procedures.

Moreover, this research revealed that perceived barriers to disciplinary communication were a crucial subject. Interestingly, time restrictions were noted by 87.50% of emergency medicine practitioners, and this has a substantial influence on the quality of treatment provided (p=0.045). This is consistent with other research showing that time constraints in emergency situations often result in hurried evaluations and a higher chance of misdiagnosis [16,17]. Furthermore, 95.59% of internal medicine practitioners acknowledged the absence of standard procedures (p=0.001), indicating a critical need for more integrated care pathways that improve coordination and communication among the specialties involved in the therapy of acute chest pain.

Study strengths and limitations

The interdisciplinary approach of this research, which includes the viewpoints of 218 medical experts from the fields of internal medicine, family medicine, and emergency medicine, contributes to its strengths in offering a thorough understanding of the treatment of acute chest pain. The mixed-methods methodology enriches the data obtained, collecting both quantitative and qualitative insights, and the large sample size improves the results' generalizability. Convenience sampling and the single-center approach, however, run the risk of introducing selection bias and limiting generalizability to different contexts. Furthermore, response bias may result from self-reported data, and causal conclusions may be limited by the cross-sectional design. In order to improve clinical practices and advance our knowledge of managing acute chest pain, future research should address these constraints. While the results provide important insights into the clinical practices and communication patterns across specialties, it is essential to note that the practice environment could significantly influence these findings. For instance, emergency medicine practitioners working in different settings (e.g., urgent care vs. hospital-based emergency departments) may have access to varying resources such as ECGs and troponin tests. This could account for some observed differences in diagnostic and management practices. The survey did not specifically capture information about the care setting, which could have provided a more nuanced understanding of how these settings impact clinical decision-making. We recommend that future studies include questions about the practice environment to further explore how the care setting influences clinical practices and outcomes.

## Conclusions

The current study underscores notable variations in managing acute chest pain across family medicine, emergency medicine, and internal medicine, emphasizing the need for a cohesive, multidisciplinary approach. It was observed that emergency medicine professionals utilized diagnostic tools such as ECGs and troponin tests with the highest frequency, aligning with the time-sensitive demands of their practice. However, significant challenges related to time constraints and communication barriers were also reported.

Internal medicine practitioners were found to have the highest adherence to management protocols, but a lack of standardized guidelines emerged as a critical barrier within this specialty. In family medicine, while strong interdisciplinary collaboration was noted, lower utilization rates of advanced diagnostic tools highlighted a reliance on referrals for further evaluation and care, likely influenced by the outpatient nature of their practice settings.

These findings indicate that standardized protocols tailored to the operational realities of each specialty, combined with improved communication pathways, are essential to optimizing diagnostic accuracy and enhancing patient outcomes. Developing and implementing a unified framework for acute chest pain management is crucial to addressing these disparities and improving care delivery across healthcare settings.

## Appendices

Questionnaire for Data Collection 

Section 1: Demographic Information

1. Age: 

[a] <30 years 

[b] 30-40 years 

[c] 41-50 years 

[d] >50 years 

2. Gender: 

[a] Male 

[b] Female 

3. Specialty: 

[a] Family Medicine 

[b] Emergency Medicine 

[c] Internal Medicine 

4. Years of Clinical Experience: 

[a] <5 years 

[b] 5-10 years 

[c] >10 years 

Section 2: Clinical Practices

5. How frequently do you use the following diagnostic tools for acute chest pain? 

   (Options: Always, Frequently, Sometimes, Rarely, Never) 

[a] Electrocardiogram (ECG) 

[b] Troponin Tests 

[c] Imaging Studies (e.g., X-ray, CT, or echocardiogram) 

6. Do you follow any established management protocols for acute chest pain? 

[a] Yes 

[b] No 

7. If yes, which protocols do you use most often? 

[a] Institution-specific protocols 

[b] International guidelines (e.g., ACC/AHA) 

[c] Informal practices 

8. How often do you perform triage and immediate stabilization measures? 

[a] Always 

[b] Frequently 

[c] Sometimes 

[d] Rarely 

[e] Never 

Section 3: Communication Patterns

9. How regularly do you communicate with professionals from other specialties when managing acute chest pain? 

[a] Regularly 

[b] Occasionally 

[c] Rarely 

10. What is your preferred method of communication for interdisciplinary collaboration? (Select all that apply) 

[a] Verbal updates 

[b] Written reports 

[c] Digital tools (e.g., EMR messages) 

Section 4: Challenges in Management

11. Which of the following challenges do you face in managing acute chest pain? (Select all that apply) 

[a] Time constraints 

[b] Communication barriers 

[c] Lack of standardized protocols 

[d] Patient-related factors (e.g., comorbidities, non-specific symptoms) 

12. How significant is the lack of standardized protocols in your practice? 

[a] Very significant 

[b] Significant 

[c] Somewhat significant 

[d] Not significant 

13. What are the most common barriers to communication in your practice? 

[a] Lack of clear communication channels 

[b] Differences in terminology or practices across specialties 

[c] Delayed responses 

Section 5: Perceived Needs and Suggestions

14. Do you believe additional training and education on acute chest pain management would benefit your practice? 

[a] Yes 

[b] No 

15. What improvements would you suggest for better interdisciplinary collaboration? 

__________________________________________________________________________________________________________________________________________________________________________

16. Would the development of unified management protocols enhance your practice? 

[a] Yes 

[b] No 

17. Please provide any additional comments or suggestions for improving acute chest pain management. 

__________________________________________________________________________________________________________________________________________________________________________

Semi-Structured Interview Guide (Optional for Selected Participants):

1. Can you describe your typical approach to managing a patient presenting with acute chest pain? 

2. How do you perceive interdisciplinary collaboration in your setting? 

3. What specific challenges do you face in following or implementing management protocols? 

4. In your opinion, how can communication across specialties be improved? 

5. Are there any systemic or institutional barriers that hinder optimal care for acute chest pain? 

6. What role do you think training and education play in improving acute chest pain management? 
